# Parents’ Perceptions on Barriers and Facilitators of Physical Activity among Schoolchildren: A Qualitative Study

**DOI:** 10.3390/ijerph18063086

**Published:** 2021-03-17

**Authors:** Vanesa Alcántara-Porcuna, Mairena Sánchez-López, Vicente Martínez-Vizcaíno, María Martínez-Andrés, Abel Ruiz-Hermosa, Beatriz Rodríguez-Martín

**Affiliations:** 1 Department of Nursing, Physioterapy and Occupational Therapy, Faculty of Health Sciences, Universidad de Castilla-La Mancha, 45600 Talavera de la Reina, Toledo, Spain; Vanesa.Alcantara@uclm.es (V.A.-P.); Beatriz.RMartin@uclm.es (B.R.-M.); 2 Department of Physical, Artistic and Music Education Didactics, Faculty of Education, Universidad de Castilla-La Mancha, 16071 Ciudad Real, Spain; Abel.RuizHermosa@uclm.es; 3 Social and Health Care Research Center, Department of Nursing, Physioterapy and Occupational Therapy, Universidad de Castilla-La Mancha, 16071 Cuenca, Spain; Vicente.Martinez@uclm.es (V.M.-V.); Maria.Martinezandres@uclm.es (M.M.-A.); 4 Faculty of Medicine, Universidad Autónoma de Chile, 3460000 Talca, Chile; 5 Department of Nursing, Physioterapy and Occupational Therapy, Faculty of Nursing, Universidad de Castilla-La Mancha, 02006 Albacete, Spain

**Keywords:** physical activity, schoolchild, parents, perceptions, attitude, focus group, qualitative research

## Abstract

Given that physical activity (PA) plays an important role in early childhood, understanding the factors that affect the practice of PA at an early age could help develop effective strategies for overcoming barriers and increasing activity levels in this age group. A qualitative study was conducted based on grounded theory aimed at exploring the perceptions of mothers and fathers from Cuenca and Ciudad Real (Castilla La Mancha, Spain) regarding barriers and facilitators of physical activity of their children during the adiposity rebound period. Data were collected using focus groups involving 46 parents of children in the 3rd grade of pre-school and 1st grade of elementary school. During the analysis, the socio-ecological model and grounded theory were used. The barriers encountered were the preferences of children for sedentary activities (individual factors), academic tasks as a main priority of parents, the influence of older siblings and the unfavorable school environment (microsystem), the lack of family conciliation (mesosystem), and barriers related to the built environment or lack of facilities for physical activity (exosystem). Facilitators were the preferences for active games (individual factors), parental models including the co-participation of parents in activities, the influence of friends, living in large homes, the support provided by teachers and the school (microsystem), living in rural areas, having sufficient facilities, favorable weather conditions (exosystem), and the existence of free or subsidized activities (macro system). Programs aimed at promoting PA in early childhood should include strategies that address contextual factors and not only focus on individual factors related to the child.

## 1. Introduction

Childhood obesity and overweight are major public health problems, and in recent years the incidence of childhood obesity has increased dramatically in some Mediterranean countries of the European Union [1], with Spain being the fourth country of the European Union with the highest rates of childhood obesity [2,3]. In Spain, the prevalence of overweight/obesity is particularly high, ranging from 19.8% to 29.4% in schoolchildren aged four to six years [4,5].

It is known that childhood obesity generates comorbidities such as dyslipidemia, hypertension, obstructive sleep apnea, type 2 diabetes mellitus, and non-alcoholic fatty liver disease in adulthood [6,7,8,9] and is also associated with poor academic performance, and low self-esteem and quality of life [10,11]. Moreover, obesity and overweight in adulthood are related to a high body mass index (BMI) during childhood. For this reason, it is essential to understand what happens at the time of adipose rebound, a normal physiological process in which the storage of reserves occurs to prepare the child for growth and development towards puberty [12,13,14,15,16]. The adipose rebound that normally occurs in children between the ages of four and seven years is a critical phase of great relevance when establishing interventions to prevent childhood obesity, and its consideration is especially important in children with early adipose rebound, when it occurs between the ages of two and five years, as this may predispose them to obesity in adolescence and adulthood [12,13,14,15,16]. As we can see, childhood is a critical period for physical, cognitive, social, and emotional development, such that presumably the interventions aimed to consolidate healthy behaviors that are going to be long-life maintained are most effective when they are implemented in this period [17].

Engaging in moderate-to-vigorous physical activity (PA) in childhood is positively associated with biopsychosocial and cardio–metabolic improvements [18] and with a reduced risk of childhood obesity [19]. In this sense, the World Health Organization (WHO) recommends that children under the age of five engage in 180 min of PA per day, of which 60 min should be of moderate to vigorous intensity [20]. For children older than five, at least an average of 60 min/day of moderate-to-vigorous intensity exercise, mostly aerobic, is recommended [21]. However, research suggests that many children do not meet these recommendations, which is associated with serious health problems in childhood and adulthood [22]. Repeated reports show that as children get older, their levels of PA decrease and the time spent in sedentary behaviors increases [2]. Although the WHO recommends avoiding periods of more than 60 min of sedentary behavior at a time [20], previous studies have shown that children spend an excessive time engaging in sedentary activities throughout the day [2,23].

According to previous studies, childhood obesity can be a consequence of the child’s exposure to an obesogenic environment [24,25], and it is known that individual behaviors are not only influenced by personal circumstances but also by social and physical environmental factors. Examining the socioenvironmental context of children is a pivotal strategy to promote healthy PA related behaviors [26].

The socio-ecological model (SEM) is a theory-based comprehensive framework useful for the analysis of the multidimensional and interactive influences of individual, social, and environmental factors that regulate behaviors [27]. This model states that determinants of PA related behavior are placed in four levels (microsystem, mesosystem, exosystem, and macrosystem), which are concentrically arranged around the individual factors of the child. Thus, the microsystem contains the elements of the closest environment to the child, such as family and school. The mesosystem includes the interrelation of two or more systems in which the person participates, such as the relationship between the family and the school or the interrelation between the family, work, and social life. The exosystem includes broader environments in which children do not have to actively participate, but in which their actions or decisions influence them. The last level or macrosystem, contemplate all those aspects related to culture, values, and legislation that indirectly also exert some kind of influence on the behaviors related to PA in childhood [27].

Despite the fact that the cultural and social context can influence the PA related behavior of schoolchildren and that the family environment is an important setting for the health promotion of children [28,29,30], little is known about the perceptions of mothers and fathers regarding the PA of their children during the transition from preschool to the school age period, which coincides with the adiposity rebound, a critical period of hormonal change [13,14,15], even though previous studies have analyzed facilitators and barriers to PA related behavior [31,32,33,34,35,36,37,38,39,40,41,42,43,44,45,46,47,48,49,50,51]. To our knowledge, only two studies [33,52] include in their samples parents of Spanish children at the age close to the adiposity rebound period.

Summarizing some of the findings found above in relation to parents’ perceptions of facilitators and barriers to children’s physical activity, other studies have highlighted that the main facilitators of PA are parental modelling [33,35,38,41,43,47,48], co-participation in activities [33,35,41,42,43,47,48], children’s preference for activities that involve movement [31,35,36,39,42,45,48], the influence of peers [31,35,36,39,42,45,48], favorable weather conditions, and living in rural settings [33,34,35,45,46,47,50,51]. On the other hand, some of the barriers identified by previous studies have been the factors related to family dynamics, workload and lack of parental time [31,33,35,39,42,43,45,48], the lack of facilities and the offer of organized activities [31,35,38,48], adverse weather conditions [33,34,35,39,42,45,46,47], and the lack of security in the neighborhood [31,33,35,42,45,47,48,49].

However, although there are previous studies that already point to what may be the determinants of PA in this period, given the scarcity of studies that have included parents of Spanish children in their samples, it was considered important to analyze the issue in this context. Therefore, this study aimed to explore the perceptions of mothers and fathers on the barriers and facilitators of PA in children at the time of adiposity rebound in Spain.

## 2. Materials and Methods

### 2.1. Design

This research is part of the MOVI-KIDS study, a cluster-randomized clinical trial aimed at assessing the effectiveness of a PA intervention on preventing obesity and improving physical fitness in children aged four to seven years old in the school setting [53,54,55], which included this nested qualitative study based on grounded theory [56,57]. We used focus group discussions (FG) to gather data from parents of 3rd year pre-school and 1st year primary school years children belonging to 24 schools from the provinces of Cuenca and Ciudad Real, Spain. This study was reported according to the Standards for Reporting Qualitative Research [58].

### 2.2. Ethical Issues

After obtaining approval by the School Council, the objectives and methodology of the study were presented to the parents of the participating children, who were asked to provide written informed consent. In addition, the verbal approval of the participating children was requested. The study was approved by the Ethics Committee in Clinical Research of the “Virgen de la Luz” Hospital in Cuenca and the Science and Education Department of the Castilla La Mancha Government, Spain (FIS PI12/02400 and FIS PI12/00761). All mothers and fathers who participated in the FGs signed the informed consent, and their participation was encouraged with a small gift.

### 2.3. Selection of Schools and Informants

After segmentation by environment (rural or urban), two schools in Cuenca and two in Ciudad Real were selected. The directors of the schools were requested to access mothers and fathers selected using theoretical sampling. The selection criteria intended to ensure heterogeneity among the FG participants, and among the groups themselves, by inviting (i) the same number of mothers and fathers; (ii) parents of different socioeconomic levels (low, medium-high, and high); (iii) different types of families (single-parent families, families with one child, or families with more than one child); (iv) workers and unemployed mothers and fathers; (v) normal weight and overweight or obese children, and vi) mothers and fathers with different levels of involvement in the PA of the children.

### 2.4. Data Collection Procedure

Focus groups were used for data collection to enable a deep understanding of the studied phenomenon [59]. Two FGs were carried out in each school between May and June 2015, one with mothers and fathers of children in 3rd grade of preschool education and the other with mothers and fathers of children in 1st grade of primary education. In each FG, the moderator had a script of topics that was adapted throughout the study and that allowed for research into the participants’ perceptions of their children’s PA (Table 1).

Ten people were invited per FG, and the participants were informed by telephone of the day, time, and place of the event. The FGs, which lasted an average of 60 min, were held in a neutral room in the schools, with adequate lighting and temperature and free of noise and interruptions. The FGs were conducted by a moderator (VAP) and an observer (LFG) and recorded on audio. The moderator and the observer were women with training and experience in qualitative research. The moderator was in charge of directing the FG, while the observer kept field notes. Neither of the two knew any of the participants or had any relationships with participants before conducting the FG. No other people apart from participants, moderator, and observer were present in the room.

Prior to beginning the focus groups, the objective of the study was briefly explained, informed consent was given, authorization was requested for audio recording, and a questionnaire was administered to collect the participants’ socio-demographic variables. The confidentiality of the participants was always guaranteed. The FGs were transcribed with the help of f4-transkript software. To ensure the accuracy of the transcripts, they were reviewed by a third researcher. Furthermore, participants were given the option of reviewing the transcripts to verify their agreement.

### 2.5. Data Analysis

Following the principles of grounded theory [56,57], an inductive analysis was performed after each FG was transcribed using the constant comparison method [60] and open, axial, and selective coding processes [61]. ATLAS-ti version 8.4.24 software (Scientific Software Development Gmb, Berlin, Germany) was used as computer support during this phase.

Data analysis was performed independently by two researchers trained in qualitative research, resolving discrepancies by consensus. The data were analyzed simultaneously, allowing the development of concepts and the theoretical saturation. The constant comparison between the codes and their relationships allowed us to go back and forth until we reached the final categorization. During open coding, the texts were organized, and the initial concepts, categories, and properties were generated. During axial coding, relationships were created, and the previous conceptualization was refined to form subcategories. During selective coding, the information was integrated until a substantive theory was formed that explained the barriers and facilitators for PA among school children [62].

## 3. Results

**Figure 1:**
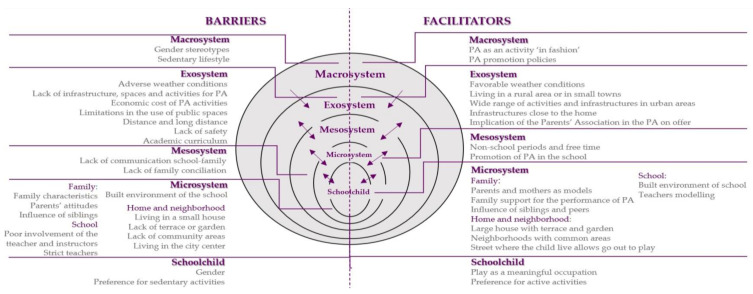
Barriers and facilitators to physical activity for schoolchildren: Diagram of categories and subcategories. Note: Adapted from the socio-ecological model.

Forty-six parents of schoolchildren (35 mothers and 11 fathers) participated in the eight FGs. Eighty parents were invited, ten per FG. Thirty-four did not attend the appointment and did not explain the reasons. Table 2 shows the socio-demographic characteristics of the participants in the study. During the analysis, we used the levels of the SEM (individual factors, microsystem, mesosystem, exosystem, and macrosystem) to organize the findings. Two main themes emerged from the data analysis: barriers and facilitators for PA among children (Figure 1).

### 3.1. Barriers to Physical Activity in Schoolchildren

Parents considered the existence of factors at all levels of the socio-ecological model that limited the physical activity of school children (Table 3, Table 4, Table 5, Table 6 and Table 7).

#### 3.1.1. Individual Factors

According to the participants, the gender of children influenced the preferences for playing activities, with girls being less active than those of boys (some girls showed a preference for more sedentary games such as playing dolls, while boys preferred sports or movement related activities). In addition, although most parents felt that young children were full of energy, some children with a quieter personality preferred spending their free time playing video games or board games, watching TV, or playing with dolls.

#### 3.1.2. Microsystem

##### Family Factors Limiting Physical Activity

Single-parent families perceived more difficulties for involving their children in after school activities or covering the costs of the same, requiring help from others during holidays, or for commuting to school. Parents also stated that unemployment was a barrier due to the financial difficulties related to paying for after school activities or purchasing sports equipment.

Although participants did not disregard the importance of their children being physically active, they assigned greater value to their academic success or learning English. In addition, as a means of punishment, they admitted to imposing restrictive schedules and forbidding their children to play or go outdoors. In some cases, they encouraged their children to watch TV to keep them entertained while they did other activities. Moreover, the gender role of parents also influenced PA. Thus, mothers who spent more time at home with their children preferred to do activities such as crafts or baking with them. Finally, the participants considered that overprotection was a barrier as they failed to allow children to go out alone on the street or commute to school, even when they lived in rural areas.

Furthermore, according to the participants, in families with older siblings, these encouraged the younger child to perform more screen related sedentary behaviors (video games, computers, tablets, or watching television).

##### Educational Factors That Limit Physical Activity

Factors related to the teachers, the school staff, or the built environment of the school could also reduce the opportunities for the children to be physically active, especially teachers with a low level of involvement in PA promotion tasks and those who only promote PA through competitive sports, ignoring recreational PA.

Moreover, participants expressed that the scarcity of facilities and their deterioration, as well as the poor arrangement of spaces (e.g., playgrounds occupied mostly by older children) could negatively affect the younger schoolchildren’s motivation for PA. In addition, some parents perceived that the schools offered few after school activities related to PA, with large differences in the activities offered between schools.

##### Home and Neighborhood Factors That Make PA Difficult

Living in small homes, which usually have no terrace or courtyard, as well as the lack of parks or public gardens in the neighborhood were considered barriers to active play.

#### 3.1.3. Mesosystem

##### Lack of Communication between the School and the Family

Most participants ignored the characteristics of the PA that their children performed within the school setting (inside the classroom, during recess, or in in physical education classes), mentioning that their children were the only source of information, and they had difficulties reporting what type of exercise they performed in physical education classes. In addition, some of the parents acknowledged that they did not usually ask their children about what they were doing in these classes, despite being interested in what they were doing in other subjects. Furthermore, the parents considered that the information they received from the school on this subject was insufficient, making it difficult to reinforce the active behavior that supposedly was being promoted at school.

##### Lack of Family Conciliation

According to the participants, the children were excessively concerned with the large amount of daily homework and after school activities, which limited the free time available for active play and other PA. In addition, they acknowledged that they had difficulty finding time to share with their families because of the excessive workload.

Having to stay at home, which generated boredom in the children, was also perceived as a barrier to PA. In addition, participants acknowledged that they lacked time to play or to perform PA with their children or take them to organized activities.

#### 3.1.4. Exosystem

##### Barriers in the Community and in the Physical and Built Environment That Made Physical Activity Difficult

According to the participants, the children were reluctant to go out and play during poor weather, preferring to stay at home performing more sedentary, screen-related activities. In addition, the lack of accessibility of leisure and sport facilities, especially in the rural environment, and the associated costs and time constraints, were considered a limitation for the PA.

The perception of a lack of security in the environment (excessive traffic, robberies, kidnappings, or accidents) limited time for active free play requiring adult or older sibling supervision. Moreover, according to parents, long distances were one of the main reasons for using the car.

##### Factors in the Academic Curriculum That Limited Physical Activity

Parents perceived that their children did not receive enough PA at school, spending long hours sitting down. Parents felt that the number of hours provided for in the regulated academic curriculum in the region of the country where they lived was insufficient. In addition, it was also negative for them that the curriculum established by the regional government considered the subject of education as examinable, as they felt that not all children had similar motor skills, which diminished their perceptions of self-efficacy and their motivation for PA.

##### Influence of the Media

Parents considered that the media encouraged the use of video consoles or tablets, contributing to sedentarism.

#### 3.1.5. Macrosystem

*Factors in the socio-cultural context that limited physical activity.* According to the parents, current lifestyles imply less PA and increased sedentary behaviors among young children, considering that they were more active in their childhood because they were allowed to go out alone, facilitating active free play. In addition, they recognized that gender stereotypes were influential, for example, the idea that certain sports activities such as soccer were "not for girls", could decrease girls’ motivation to engage in sports that are traditionally associated with boys. Finally, the aggressive behaviors exhibited by some parent’s during sports competitions negatively influenced the decision to enroll their children in certain activities.

### 3.2. Physical Activity Facilitators for Schoolchildren

Table 8, Table 9, Table 10, Table 11 and Table 12 shows the main facilitators for school PA along with a synthesis of the participants’ verbalizations. 

#### 3.2.1. Individual Factors

*Individual facilitators that encourage physical activity.* Participants felt that young children were very active and that games involving movement were important in childhood. In addition, their children continuously demanded movement games, both indoors and outdoors; moreover, participants mentioned that their children preferred games that involved movement, often demanding to go to the park or the street to play with other children.

#### 3.2.2. Microsystem

##### Family Factors Promoting Physical Activity

The parents were conceptualized as a model for their children, considering that if they maintained an active lifestyle, it was easier for their children to be active as well. Participating in activities and sharing time with their children were seen as powerful facilitators of PA for their children. Families encouraged activities in nature, sports, biking, and active play in general, especially on weekends and holidays. The participants emphasized that most fathers practiced some kind of sport with their children, while most of the mothers shared more play time with their children at home. Furthermore, the parents usually respected their children’s preferences for after school activities, usually sports activities. In addition, the parents were aware that PA in childhood is very important because of its physical, psychological, and social benefits. Finally, having siblings, especially those of similar age, also stimulated indoor play, helping the children to be more active.

##### Influence of Peers

According to participants, young children were more active when they were with peers, often demanding to play with their friends on the street. In addition, friends influenced the afterschool activities chosen by the children.

##### School Factors Promoting Physical Activity

Recess was an ideal period to engage in activities that involved movement. In addition, most participants felt that teachers facilitated the involvement of children in PA since they acted as role models. In addition, they felt that their children returned home happier when the activities in the physical education subject encouraged play, compared to when they practiced a particular sport or just ran. In addition, appropriate school facilities helped provide the necessary materials to carry out the activities and help keep their children active.

##### Household and Neighborhood Factors That Encouraged Physical Activity

According to the participants, living in large houses, single-family homes, or homes with a terrace or garden, and neighborhoods with common spaces facilitated children’s PA. Parents who lived in housing estates within cities reported that their children were more active than children who lived in flats without common areas or flats, as they allowed their children to go outdoors alone within the estate to play. Participants who lived in a village, where single-family houses with large spaces to play in are more common, also felt that this type of housing facilitated their children’s physical activity, compared to those living in flats or flats. Participants also felt that living on a street with low traffic or a pedestrian street made it easier for children to go out and play with their friends.

#### 3.2.3. Mesosystem

##### Holiday Periods and Free Time

On holidays or weekends parents encouraged children to be more active by having more time for active play with peers. According to the participants, they often took advantage of these periods to travel, go on excursions, and do outdoor activities. Parents pointed out that many weekends and holidays were used for activities with other family units, or even participated in activities that allowed them to meet other people, which facilitated their children’s socialization. They also emphasized that these were ideal times for children to spend time with other family members such as grandparents, aunts, uncles, and cousins.

##### The School as a Setting for Conveying Values

According to the participants, the school was the best place to promote PA among children, as the after-school activities aimed at increasing the activity level of their children were perceived as being positive. Parents saw as very positive the activities that schools carried out in coordination with associations and town councils to organize tournaments or charity races. In addition, they indicated that the school often organized sports activities in which they themselves could participate with their children, thus facilitating participation in intergenerational activities.

#### 3.2.4. Exosystem

##### Community Facilitators and the Physical and Built Environment Promoting Physical Activity

Good weather, having adequate sports facilities, especially in urban areas, having an adequate offer of organized activities, and environmental infrastructures for active commuting were the main facilitators for PA. In addition, living in a rural area or in a small city was a facilitator, as was parents allowing their children go out to play in the street and walking.

##### Organization of after School Activities Related to Physical Activity

Participants felt that the parents’ association facilitated children’s PA by organizing most of the after-school activities in the schools. In addition, they felt that if the parents’ association organized more sports activities it would help promote children’s PA.

#### 3.2.5. Macrosystem

*Facilitators of the socio-cultural and political context.* Parents considered PA to be socially fashionable. Thus, the fact that children saw people running in the street or advertisements promoting sports materials could positively influence their PA. In addition, the fact that activities and spaces related to PA were totally or partially subsidized by the municipality was another facilitator.

## 4. Discussion

Mothers and fathers perceive barriers and facilitators to PA for school children at all levels of the socio-ecological model. The facilitators of PA included children’s preferences for active games, parental modeling, parents’ co-participation in activities, the influence of peers, living in large houses, support from teachers and the school, living in rural areas, having sufficient infrastructure, favorable weather conditions, and the existence of free and subsidized activities. Conversely, the main barriers were the increasing preferences for screen time, the parents’ concerns for academic success, the influence of older siblings and the unfavorable school environment, the overload of parents and schoolchildren, and the physical barriers related to the school and neighborhood for active commuting.

About the individual factors and in line with previous studies [31,33,35,39,44,51], the parents considered that young children are very active and full of energy, which led them to believe that children’s PA is high because they are constantly on the move. However, this belief contrasts with studies that point out that young children do not meet daily recommendations for PA [20,63], and that at home they tend to spend most of their time sitting or doing light-intensity activities [23]. This could be because parents are not able to objectify the amount of PA their children do. Following these results, it would be interesting if parents had tools to objectively measure the amount of PA that their children do during the day. However, most parents are unaware of the PA recommendations for young children. Future research should study this aspect in order to determine the causes of this lack of knowledge and promote strategies involving both children and families, as training and raising awareness among parents along these lines could increase the level of daily PA performed by children.

However, it is known that play is an activity that is highly valued by children, being perceived as a pleasant occupation that can directly influence their well-being and happiness [64], and that participating in PA contributes to the satisfaction and improvement of the performance of daily activities [65]. Our results confirm these findings, showing that young children prefer to engage in activities related to PA or active play, with play being a significant occupation in childhood. According to our findings, young children prefer active play and playing with other children outdoors, relegating quiet or sedentary play to when they cannot go outdoors or have no one to play with. These results are consistent with a recent study [66] that showed a positive association between time spent outdoors with increased PA and decreased sedentary behaviors. Thus, each additional hour that children are outdoors is associated with an increase in the amount of light and moderate-to-severe PA [66]. Therefore, encouraging young children to go outdoors and play could increase their daily PA. Despite this, some studies point out that after school, children spend a large part of their free time sitting down [67,68].

In line with previous studies [68,69,70], our results also show that there are differences between boys’ and girls’ preferred activities, although during the FG, most of the parents believed they facilitated that there were no gender differences in the performance of PA. In their discourse, it was evident that, when it came to choosing activities, girls preferred more active and sedentary activities and games, such as playing with dolls or board games, while boys preferred activities that involved more movement, such as playing with the ball. Their discourse also reflected that there were differences in their children’s choice of extracurricular activities, with girls often participating in activities related to dance and rhythmic gymnastics, while boys preferred activities such as karate and football.

As has been pointed out in another study [68], perhaps this difference in the choice of the type of activity may be conditioned by the range of extracurricular and sporting activities on offer in the area where the children live or by the process of enculturation and socialization of the children. Following on from the above, the participants who lived in rural areas felt that the range of activities on offer in these areas was more limited than in the cities and that the activity to be carried out par excellence was football, a sport traditionally considered to be a boys’ sport. In this sense, parents felt that they were happy for their daughters to play football or take part in traditionally male sporting activities, supporting them when they demanded it. However, they also mentioned that nowadays, the activities that girls do are still conditioned by gender stereotypes, where some sport activities are still considered masculine and this can limit their participation in them.

In line with previous studies [43,69], these differences in activity preferences between boys and girls may also be due to the tastes and interests of mothers and fathers. Participants noted that mothers are traditionally more involved in maintaining the house and the family, while fathers, in their free time, take the opportunity to go outside with their children to ride bikes or play outdoors. In line with other studies [33,35,38,41,43,47,48], parental modelling and co-participation may play a key role in generating new interests that influence children’s PA behaviors. Future research should further analyze the differences between boys and girls in terms of their preferences for PA-related behaviors.

In relation to above and after analyzing the factors that can be included in the microsystem and mesosystem, our results show the great influence that parents and teachers, as well as siblings and friends, can have on the amount of daily PA that children perform. In this line, it is important to highlight that these social agents can act as facilitators or barriers to children’s PA at the same time. In this sense, parents’ perceptions are in line with previous studies that report that social and family support are facilitators for PA [33,35,38,41,43,47,48]. When parents have a positive attitude toward PA, children are more physically active [70]. Furthermore, although the built environment and the existence of green spaces influence the level of PA in school children [33,34,35,45,46,47,50,51,71], parental support has a greater influence on their PA [71].

Moreover, despite the fact that parents attribute multiple health benefits to the practice of PA [34,35,40,45,46,49] when choosing activities, parents tend to prioritize those linked to academic achievement. Furthermore, although there is strong evidence confirming the positive relationship between PA and academic and cognitive performance [10,11,72,73], participants do not perceive that PA improves learning or school achievement. Therefore, raising parental awareness of the positive relationship between PA and academic performance may help promote children’s PA.

Furthermore, our results coincide with previous studies that confirm that difficulties of family conciliation and parental over-occupation [31,33,35,39,42,43,45,48] limit the level of PA of children and their time for outdoor play. In this respect, most of the working parents reported that they hardly had time to play with their children or participate with them in PA between the day-to-day activities. This problem was also accentuated when both mothers and fathers worked, in cases of single-parent families, who depended on the help of others for childcare or when parents worked far away from the family home. When parents perceive difficulties in getting their children to do PA regularly, such as considering that taking their children to afterschool activities is an extra workload, the probability that they will choose these activities decreases [74]. In line with other studies [31,33,35,37,42,45,48,75], our results reflect that the perception of excessive workload encourages the use of the car, even in rural areas where distances are short. Future research should explore this further.

In relation to the above, our results show a trend toward child over-occupation, which reduces the time spent by children in PA or active play. A previous study shows the permissiveness of parents regarding their child’s inactivity as long as they do activities with educational value, differentiating between activities that are beneficial (doing homework or reading stories) and not beneficial (watching TV or playing video games) for children [76]. Our results follow this line, showing the tendency of parents to limit screen viewing time, considering that its excessive use is harmful [45,48,49]. This study also shows that the final decision on the type of activity performed by young children falls on the parents. However, although our results show that parents respect their children’s preferences for active play, according to another study analyzing children’s perceptions, this is not the case [68]. In our study, this discordance may be due to the high number of unemployed people among the participants. In line with other studies, our findings show that the cost of organized activities can limit the PA of school children [31,33,35,39,42,48] as can the lack of free or subsidized spaces in their places of residence.

In terms of peer support, in line with other studies [31,35,36,39,42,45,48], our results show that support among siblings and friends can increase the PA performed by school children. However, we also found that the presence of older siblings may facilitate sedentary activities. In addition, the results show that, as children age, they spend more time sitting, which may be due to changes in their daily occupations, marked by an increase in school demands or a change in their preferences regarding types of activities. Future research should confirm these issues.

In relation to the exosystem and macrosystem, it should be pointed out that our results are congruent with previous studies that describe that aspects of the built environment, such as the lack of parks and sports facilities, or the lack of appropriate spaces within the school are barriers to PA [77,78,79,80]. Therefore, improving accessibility and environmental infrastructure and promoting active commuting [81] may influence the activity levels of young children. Furthermore, during the FGs many of the parents expressed that the cost of after-school activities was a real problem for them. This discourse was most recurrent in the case of low-income or unemployed parents. Many of these parents pointed out that they could not sign their children up for the activities they wanted. In their discourse, they expressed the need for their local council or government to offer more activities free of charge or to support them through subsidies to facilitate their children’s PA.

## 5. Strengths and Limitations of the Study

The strengths of this study include the methodological rigor, reliability, and validity of data through verification strategies and continuous reflexivity [82]. The FGs were immediately transcribed and their preliminary analysis enabled the refinement of the focus group script. The process of selecting participants and theoretical sampling enabled the rapid detection of key informants and reaching theoretical saturation. Data and researcher triangulation strategies were followed, and participants were given the option of reviewing transcripts. Data were transcribed verbatim. In addition, this study followed the recommendations of the COREQ statement [83].

Several limitations must be highlighted. Thus, there was a low representation of men among the participants, together with a high number of housewives and unemployed people. The heterogeneous composition of the FGs was a further limitation. Future studies should analyze this phenomenon by including homogeneous samples or by performing segmentation according to factors such as place of residence or socioeconomic level.

## 6. Conclusions

Young children prefer activities that involve active movement and play; however, the final decision on the type of activities undertaken is conditioned by the beliefs, preferences, and burdens faced by the parents. Friends and siblings, especially those of similar age, promote PA, although older siblings may also promote sedentary activities. Parents consider that the school has a fundamental role in the promotion of their children’s PA; however, the lack of involvement of teachers and the scarcity and poor state of infrastructure are limiting factors. In addition, the characteristics of the physical and built environment, traffic, and the perception of lack of safety of the environment limit the PA of schoolchildren aged 5 to 7 years.

It is necessary to implement policy guidelines that ensure the presence of free spaces and infrastructure or subsidized organized activities, as well as family reconciliation measures.

## Figures and Tables

**Table 1:** Script of topics used in the focus groups.

Topic	Subtopic
Mothers’ and fathers’ experiences of physical activity	Regular physical activity. Physical activity practice during holidays. Self-perception. Motivation for physical activity. Value attached to physical activity
Fathers’ and mothers’ perceptions of their children’s physical activity	How time is spent. Type of activities carried out at home, in the community, and at school. Leisure versus work time. Meaning of being active versus being sedentary. Children’s self-management and attitude towards the type of activities undertaken. Parents’ expectations of their children’s activities. Determining factors in the choice of type of activity. Importance attributed to the activities carried out by the child throughout the day.
Family dynamics	Family factors that facilitate children’s physical activity. Family factors that hinder children’s physical activity. Type of activities carried out in the family. Parent modelling.
Facilitators and barriers of the physical and social environment as perceived by parents	The domestic environment: use of private space. Use of public and community space. Built environment and accessibility. Availability and use of public and private resources in the place where they live that promote physical activity.Perception of safety in public/private space.Influence of weather conditions.Social relationships and extra-familial influence.
Travel and type of transport	Places where the child usually travels. Active travel (on foot or by bicycle). Mode of transport used. Preferences in the choice of type of transport.
Material resources related to physical activity	Preferences in the use and availability of games, toys and sports materials. Children’s wishes versus parents’ wishes. Clothing and footwear. Factors influencing the purchase of equipment.
The role of the school in the physical activity of schoolchildren	Knowledge about the functioning and policies of the school. Perception of the involvement of the teaching and management team in promoting physical activity among children. Involvement of parents in decision-making about school activities. Level of satisfaction with the activities carried out by children at the school. Range of activities offered and management of extracurricular activities.
Strategies to promote physical activity among schoolchildren	Strategies parents are aware of to keep children active. Role of different social agents/responsibility for children’s physical activity. Areas for improvement at school, at home and in the community. Influences of policy, media, advertising, and marketing. Parent training strategies. Parents’ motivation as change-makers.

**Table 2:** Socio-demographic characteristics of the participants in the study.

Participant´s Characteristics	Cuenca	Ciudad Real	Total
	*n* = 25	*n* = 21	*n* = 46
**Age (years)**			
<35	4	2	6
35–45	20	16	36
>45	1	3	4
**Gender**			
Male	3	8	11
Female	22	13	35
**Area**			
Rural	11	13	24
Urban	14	8	22
**Educational level**			
No education	1	0	1
Primary education	6	9	15
Secondary education	2	5	7
Intermediate vocational training	2	1	3
Higher vocational training	0	1	1
High School diploma	3	4	7
University studies	11	1	12
**Civil status**			
Single	0	1	1
Married	23	16	39
Divorced	2	2	4
Unmarried	0	1	1
Widowed	0	1	1
**Employment status**			
Employee	14	8	22
Unemployed	11	13	24
**Profession**			
Administrative	2	0	2
Bank clerk	3	0	3
Environmental agent	1	0	1
Commercial agent	0	1	1
Real estate agent	1	0	1
Construction worker	0	1	1
Housewife	10	7	17
Self-employed	0	1	1
Teacher assistant	0	1	1
Hotel and catering professional	0	4	4
Teacher	3	0	3
Health professional–social worker	3	1	4
Sports worker	0	1	1
Civil servant	2	0	2
Pensioner	0	1	1
**Family unit**			
Mean number of children	2.28	2.24	2.26

**Table 3:** Individual factor barriers to physical activity for schoolchildren.

Individual Factors			
Categories	Subcategories	Code	Parents’ Verbalizations
Individual factors of schoolchildren that hinder their physical activity		Gender preferences	“And then the girls, they like dolls more” (M4, FG1). “I think that what they usually like the most is what she says, if they are girls they like dancing, if they are boys they like karate or football” (M3, FG2).
	Schoolchildren’s preference for sedentary activities	Quiet personality of schoolchildren	“...And the other one if it were up to her she would be lying on the couch all day, so I have to force her...” (M4, FG6). “...What happens is that, well, every child is a different world, so of the five that I have, none of them have turned out to be sporty. Since they were little I’ve been trying, but in the end, most of them have sedentary habits, at least three of them, and even if you’re on top of them and so on, it’s complicated, no, because no, not genetically, I don’t know, they must inherited it from their grandmothers” (F5, FG6). “...He’s more into books, he’s not into sport. You tell him let’s go running and pick up a book and he picks up a book” (M7, FG6).
		Children’s boredom	“Mine, she never knows what to play, they have everything, in the old days when we were little we didn’t have anything, they have video consoles or tablets or I don’t know what and all of a sudden as she tells you that she’s bored, that she doesn’t know what to play. Especially in the cold winter months. Now less so, because as soon as that happens, she says ’I get bored, I don’t know what to play’” (M1, FG1). “A lot of times they get bored, ’Oh, mum, I’m bored, I want to play I want to... let me have your mobile phone to play with the games on the mobile!’" (M2,FG8).
		Preference for playing with mobile phones, computers, or video	“The first thing they do when they come into the house is pick up the tablet, sit on the sofa and put the TV on, even if they don’t watch it, but they put the TV on and the tablet. In winter especially, now they are more inclined to go downstairs to play and all that, but in winter it’s the television, and video games, the phone, the computer and so on” (M1, FG1). “...Everything, but come on, he also likes to play video games, if you leave him the tablet, the tablet, whenever I leave it, it also takes up his time and he likes it. When he sees the mobile phone lying around, he immediately goes to pick it up, to play games” (M7, FG7). “...And then a lot of video game consoles, we just... all the video game consoles they want and more, and that’s it” (M2, FG4).
		Preference for watching TV	“...They get hooked and can spend two hours watching TV, which is nothing, and maybe they arrive just on time, and they want to continue watching TV at my house, but if you’ve been watching TV for two hours, I mean, as soon as you give them a little bit of TV, I notice that, I know, that it’s addiction to TV” (M5, FG1).

Abbreviations: M = Mother; F = Father; FG = Focus Group. Distinction of FG by rural or urban setting: Rural area: FG3, FG4, FG7, and FG8; Urban area: FG1, FG2, FG5, and FG6.

**Table 4:** Microsystem barriers to physical activity for schoolchildren.

Microsystem			
Categories	Subcategories	Code	Parents’ Verbalizations
Family factors limiting physical activity	Family characteristics	Single-parent families	“I think we also have to differentiate which family group the child belongs to. Because there are four of us here, two of them are happily married, living with a couple and the other two are not, so I think the child, specifically in my case, I think the child demands both ways, don’t you? (M4, FG2).
		Need for support from others in caring	“I think that yes, when parents are working, if you leave the person in charge, they can’t take them or bring them or go out with them, of course they do less physical activity than if you are with them” (M2, FG2).
		Unemployed parents	“...There are many unemployed parents and I would also sign him up to I don’t know what, but that’s worth a lot, you can’t afford it” (F3, FG5).
	Parents’ attitudes	Promoting academic achievement	“...’What did you do in Mathematics?’, or ’Did you have an exam?’, but if he tells me that he is going to take an exam in Physics, Physical Education, I don’t ask him ’How did you do?’ My child has got a ’B’ in gymnastics in all 3 assessments, well, and this time he says ’mummy, I got an ’A’’, and I say ’you have to get it in Maths or Spanish’, you know? yes, well, you have to get it in Spanish, because if you don’t get it in Spanish or Maths or a subject like that, I tend to get more angry” (M3, FG8).
		Parents decide what activities children do	“What I was saying is that they are induced, that right now if the mother says you go and do this, more than 7% will do it, because they will do it because, if the mother has said so, they will do it” (M2, FG5).
		Mothers encourage quiet play and sedentary activities	“I don’t, I do activities because I like baking a lot, so if I think, well, let’s make a sponge cake! and in that, yes, they get involved, they help me, I pour the flour, I give them the mixer, I... but I start playing with them, so I say let’s play, no, no” (M1, FG2).
		Overprotection	“...I’ve got this kid too groggy this boy should know how to walk on his own, he could even go to school on his own at the age of eight [...] however, because you have them so overprotected, it’s always ’I’ll take you, I’ll do it, I’ll...’, in the end they don’t know how to do anything on their own or how to go anywhere on their own” (M5, GF4).
		Use of the car	“...we’ve got used to it, and it would take five minutes to walk from my house to here. But I bring my car with me because I have to go to work afterwards, so I don’t want to waste another five minutes to go home and take the car. So I come with the car and the children and that way I can go straight away” (F1, FG7).
	Sibling influence	Older siblings encourage sedentary activities	“They have too much, so they spend too much of their free time [doing sedentary activities], they have finished their school and extracurricular activities, and they prefer to be, even with their siblings or whoever, spending their time on all these technologies...” (M6, GF4). “He has an older brother and when he was one year old he already liked to sit with his brother and watch him play, at one year old, so... when he was two years old he already knew how to use it. It’s something he loves” (M5, FG4).
Educational factors that limited physical activity	Factors related to teachers and instructors that hinder physical activity	Poor involvement of the teacher or instructor	“The other teacher is more like ’come on, do a round’ and they run around. I mean, it depends a lot on the teacher they have, it all depends, I mean, I think that he is the one who moves the class and leads it, and if he doesn’t get involved, the children won’t get involved, that’s clear” (M1, FG2). “...When a monitor, clearly has many more students and maybe he/she is not so dedicated, spending individual time with them, I mean, really, they are like I don’t want to go, I’m bored, I don’t do anything, the teacher doesn’t pay attention to me...” (M3, FG2). “...She gave up modern dance. She didn’t give it up because she didn’t like it, but because of the teacher, who didn’t motivate her” (M2, FG7).
		Strict teachers	“...The teachers should be chosen by the children, because in many cases they are very strict, and the children go to class with fear, and it shouldn’t be like that...” (M3, FG5). “...My daughters go because they like it, and they don’t care if I yell at them, if I put them last, you know? They like it. But there are girls who maybe... They aren’t warm enough, and they don’t have any tact for them...” (M4, FG7).
	Elements of the school’s built environment that hinder physical activity	Poorly maintained facilities	“...Well, apart from what she has said, to have facilities, I think in good conditions, because sometimes schools don’t have good facilities for them to practice sport” (M4, FG7).
		Scarcity of facilities	“There are only two playing fields, and they are for each day, for a different year [...] they can’t just arrive and say, today I’m bringing the ball, because it’s not their turn” (F6, FG3).
		Organization of the facilities	“It’s just that here at the school they also have a timetable for the courts, they can play football or basketball on the day they have a court available” (F6, FG3).
Home and neighborhood factors that hamper physical activity		Living in a small house	“...The type of game they play at home, depending on the fact that we don’t have much space, is more sedentary than when they go outside... in my house we don’t have much space either, like to run a lot, because you get dizzy [laughs], not so much now they are not so big” (M1, FG2).
		Lack of a terrace, courtyard or garden at home	“...It has nothing to do with having a playground with a basketball hoop for example, that you have, I don’t know, from having a lot of space, to having a very small house, a child who has to have 19 shelves because the toys can’t even be on the floor, at hand...” (M4, FG2).
		Lack of common areas in the neighborhood	“...if you have a villa in La Moraleja, with tennis fields, swimming pool, and everything, well, you see, that person is more likely to do sport than someone who lives in an 80-metre flat” (M1, FG5).
		Living in the city center	“... if you live in the center, in a flat as in this case, then those children in winter are indoors or are at home with their parents” (M4, FG5).

Abbreviations: M = Mother; F= Father; FG = Focus Group. Distinction of FG by rural or urban setting: Rural area: FG3, FG4, FG7, and FG8; Urban area: FG1, FG2, FG5, and FG6.

**Table 5:** Mesosystem barriers to physical activity for schoolchildren.

Mesosystem			
Categories	Subcategories	Code	Parents’ Verbalizations
Lack of communication between the school and the family		Children do not tell their parents what they do in physical education class	I ask my daughter ’what did you do today in gymnastics?’ and ugh... they don’t tell me anything specific, I don’t know... I’m a bit lost with gymnastics at school, especially in young children. When I was young I used to wear a tracksuit three days a week or I don’t know what the days were but they never did gymnastics” (M1, FG3).
		Parents do not ask their children what they do in physical education	“Because I... I would ask him about the classes, ’What have you done in class?’, and he would say ’well look, we have learnt this or that’, but in physical education I have never asked him ’What have you done in physical education?’, he does say ’well today mum we have played tag or this’, because they are new things that they don’t... but they don’t... they don’t talk to me about anything else, I don’t know what they call the things they do” (M4, FG8).
		Lack of knowledge regarding the activities they carry out at school	“Because neither... as you can’t see them, of course, you can’t know... and that is what... or if they have an indoor sports facility day, because of the weather, I don’t know what activities they have there in the indoor sports facility, I just don’t know” (F6, FG3).
Lack of family conciliation	Overscheduled children	Excess of homework	“...It’s that homework has enslaved them [...] they already have a working day, as young as they are, of many hours” (M4, FG1). “But I had to take them out of basketball, because they either went to basketball or they did their homework, or one thing or the other. They didn’t get to everything on time” (M4, FG5).
		Excess of organized after school activities	“...Those who have music school, music school takes up five or six hours a week, so they have the music school. So I have two children at the language school and with that they are already overworked” (F5, FG6). “Because of course, right now there are five days with activities, there are days, every day there are two activities except Friday with one, and it’s impossible, but not because I don’t want to” (F1, FG7).
		Lack of time for free play	“They have all the day taken, let’s say, and the little play they have is almost when they have to go to bed, at least my children” (F6, FG3). “I think that children outside of [...] between school, classes and everything, they don’t have time to enjoy themselves” (M4, FG5).
	Overburdened parents	Excessive workload	“I try to compensate a bit for the time I can’t spend during the week [...] But during the week I can’t spend time with her for playing, I can’t spend time dedicated to her, for example” (M8, FG6). “Many of us work so of course, you try to go with enough time, because if I take the child in a rush, I have to turn around again, so we have the same rhythm, not all of us, but at least in my case, I go with just enough time from one thing to another, so I can’t allow myself to take time out and say I’m going to walk, because I need that time for something else, so it’s not like that. Some people use the car for convenience, that’s true, but for me, at least in my case, it’s because of my work” (M2, FG3).
		Caring for several children	“But of course, it is also based on the fact that I have a young one and then I have the other older one, so of course you have to distribute...” (M7, FG6).

Abbreviations: M = Mother; F = Father; FG = Focus Group. Distinction of FG by rural or urban setting: Rural area: FG3, FG4, FG7, and FG8; Urban area: FG1, FG2, FG5, and FG6.

**Table 6:** Exosystem barriers to physical activity for schoolchildren.

Exosystem			
Categories	Subcategories	Code	Parents’ Verbalizations
Barriers in the community and in the physical and built environment that hamper physical activity		Adverse weather conditions	“...Well, we have it divided into two seasons, winter and summer. In winter it’s cold and we can’t go outside, so all the activity is concentrated inside the house” (M2, FG2). “When it rains or whatever, they go inside and play video games or whatever, or watch TV. And when you leave them, they spend all day playing video games, it’s like that...” (M1, FG4).
		Lack of spaces and infrastructures for physical activity	“...In a big city you want to practice hockey and you can practice it, you want to practice thousands of sports and you have to... maybe you have to move, because of course, you are not going to have it in your neighborhood. But here, many sports, you can’t even practice them because you don’t have the possibility to practice them, nor do you have sports facilities to do so” (F2, FG1). “And then there are no sports facilities either, because they are, let’s say, owned by the council, you have to be registered to go and play, even in the few parks, unless it’s a sports center where everyone can go and play, not here, here if you go to the football pitch you have to be registered, if you go to Judo you have to be registered, but a sports center where kids can go and play and there is no traffic at all, there is none of that. There, they have basketball courts, tennis courts, paddle courts, indoor football courts, but here, there is none of that” (F6, FG3).
		Low offers of organized activities and sports, cost of organized activities and materials	“...Maybe you have to move, because, of course, you won’t have it in your neighborhood. But here, many sports, you can’t even practice them because you don’t have the possibility to practice them” (M1, FG1).
		Cost of organized activities and materials	“What happens is that it’s more expensive and it got to a point where I said ’no’, because you have to pay for the belts, which cost a lot of money. What it costs... to go to karate, then the outfit, then the trips that you have to take to the competition... and I’m just saying no” (M2, FG8).
	Limitations on the use of public and community spaces	Restrictions and prohibitions	“You can’t play ball because it’s a park. You can’t ride a bike because it’s a park, parks have always been made for children to play, not to be restricted, right? Or removing them... Both here in the village and in a city, I know what happens, don’t I? Instead of creating free zones or clean zones for them to play in, they limit them” (F6, FG4).
		Occupation of parks and other spaces by older children	“There are also older kids, younger kids get kicked out right away and all that…” (M6, FG4).
	Long distances	Difficulty in attending organized activities	“...My girls wanted to go to gymnastics, which they do. But since it’s so long, it’s impossible. It takes them a long time to eat and they arrive at four o’clock, it’s impossible, I don’t have time” (M5, FG3).
		Promotion of car use	“...When they go to the swimming pool, they go by car because it’s a bit further away” (M1, FG1).
		Difficulties going outside to play with friends and schoolmates in urban areas	“...The after-school activity you had was to go out to the little square next, or to the park next to play, alone with friends from the neighborhood, but there was that, there were friends from the neighborhood, but now there aren’t any. Because now many of the children who come to this school don’t live in this neighborhood...” (M1, FG1).
	Perceived lack of safety	Heavy traffic	“I remember when I was little, of course it was a long time ago, but you would go out and spend the whole afternoon playing and there was no problem at all. Your mother would call you to come in for a snack and you would come in, now the kid, I live practically opposite the school and you have to take him almost up to the door, because of the traffic. Everyone goes very fast with cars up and down the street, it’s not that you don’t want him to go out, but if it’s a street, more or less a busy one, you just can’t relax” (P1, GF5). “The limitations are set by us, because even though I have a school and so on, I don’t feel safe... for the child to go out alone at the age of seven, I don’t feel safe, maybe I’m paranoid, but there is a lot of traffic and I don’t feel safe. So for the child to go out alone I don’t...” (M3, FG7). “You can’t be sure that, if you go home to get something and she’s out, you can’t, you can’t, because with the traffic alone it’s impossible to leave her alone at any time, because in any situation they arrive and it’s impossible, it’s impossible, to be aware, no, no” (F6, FG1).
		Fear of accidents and abductions	“To be happy like before, you have to go back a few, a few good years, when children could go alone to their grandmother, without fear of being stolen or stepped on, of being caught out there, just like leaving the door open, that anything can happen...” (M2, FG5). “...I don’t know what generates fear, but it is true that you are afraid that they are on the street for fear of being abducted or for fear of being run over by a car or for fear of a lot of things...” (M5, FG4).
		Need for supervision	“I don’t leave them anywhere unsupervised, I don’t leave them anywhere unsupervised, maybe I don’t know... maybe there are people who think that at seven years old they are old enough make a life for themselves” (M2, FG7).
Factors in the academic curriculum that limit physical activity		Insufficient time allocated to physical education	“I think that the role of the school in the physical activity of the children is minimal, they do the minimum, that is, there are three hours of class, of gymnastics, but they do the minimum. They don’t teach them, they don’t teach them to play basketball, or to play any sport” (M1, FG3).
		Physical education is assigned less importance than other subjects	“...But of course, if the academic obligation doesn’t leave you time for that... well... the academic obligation is a priority, whether we like it or not, this is the system. As long as they don’t give more importance to physical education” (F1, FG7).
		Physical education as a graded subject	“I think that, for example, the fact that physical education is graded can be an incentive for some children and a demotivating one for others” (F2, FG6).

Abbreviations: M = Mother; F = Father; FG = Focus Group. Distinction of FG by rural or urban setting: Rural area: FG3, FG4, FG7, and FG8; Urban area: FG1, FG2, FG5, and FG6.

**Table 7:** Macrosystem barriers to physical activity for schoolchildren.

Macrosystem			
Categories	Subcategories	Code	Parents’ Verbalizations
Factors in the sociocultural context that limit physical activity	Sociocultural conditioning	Behavior of other parents	“So I don’t understand how a parent who is of a certain age can start insulting 10 year olds, or the referee or the coach, or tell them how they are doing things, right or wrong, when they are just training, or playing football” (F2, FG1).
		Gender stereotypes	“Certain sports are still boxed in certain sexes, my daughter plays football and some mothers when you say ’I’m not going to take N to football’, ’but she’s going to football and she’s so beautiful!’" (F2, FG6).
		Lifestyle changes	“Yes, but now the activity has changed a lot, because as he says, it’s true that we used to walk everywhere, my father couldn’t take me, I didn’t have a car, and we went out shopping on foot and we went everywhere. And then we went out into the street a lot to play, because back then there wasn’t that traffic, but it’s not that there wasn’t fear, let’s see, the fear we have now is maybe because there are reasons, because more things happen now than back then” (F5, FG4).
		Influence of the media on the use of technologies	“Well, it’s the same thing, they have so much technology, that if they are not on their mobile phones all day with WhatsApp, if they are not with the PSP, if they are not with the PlayStation... they don’t know how to do anything else. Not because they don’t know how to do anything else, they do, because you teach them, you tell them to do it, but they prefer to do that. Because what they’ve been taught, not by their parents, but by everyone, the state, TV and everything, because they show it to you on TV” (F7, FG4).

Abbreviations: M = Mother; F = Father; FG = Focus Group. Distinction of FG by rural or urban setting: Rural area: FG3, FG4, FG7, and FG8; Urban area: FG1, FG2, FG5, and FG6.

**Table 8:** Individual factor facilitators for physical activity among schoolchildren.

Individual Factors			
Categories	Subcategories	Code	Parents’ Verbalizations
Individual facilitators that encourage physical activity		Play as a meaningful occupation	“...So they start to play, but we go very quickly. If they can go out in the street with a ball and run, they do it...” (M5, FG1). “They love any game, no matter what it is. Now because football is the most famous, the most, but he doesn’t mind playing...” (M4, FG1).
		Young children are very active	“...He can’t sit still, he’s a very restless child and he can’t, he can’t, I’m sitting still here” (M3, FG2). “He is very active and spends all day playing” (F2, FG6).
	Schoolchildren’s preferences	Preference for after school activities linked to physical activity	“Practicing any type of activity, anything that involves moving, seems very appropriate for him. So football, swimming... whatever, anything that involves movement” (M3, FG7). “Well, he prefers Movi-Kids to English, he prefers it” (M4, GFG8).
		Preference for active free play	“...In my house she dances from the moment she gets up to the moment she goes to bed, it’s incredible, what she likes, so of course, in my house she’s there all day long too. And then if we go to the park for a while in the afternoon, there are days when she takes her skates or her bicycle down...” (M4, FG7).
		Preference for going outdoors to play	“He always wants to go out, well, if it were up to him, we’d be there all afternoon from six o’clock and he’s already saying “well, when are we going to go? When are we going to go?”, he likes it more... And then... I mean, if I don’t go out he goes out, it’s more about going downstairs than getting on the machine, he likes it more” (M1, FG4).

Abbreviations: M = Mother; F = Father; FG = Focus Group. Distinction of FG by rural or urban setting: Rural area: FG3, FG4, FG7 and FG8; Urban area: FG1, FG2, FG5 and FG6.

**Table 9:** Microsystem facilitators for physical activity among schoolchildren.

Microsystem			
Categories	Subcategories	Code	Parents’ Verbalizations
Family factors that promote physical activity		Parents as role models	“If they have never seen it at home or if you see your parents sitting there all day doing nothing, they follow the same... routine as their parents, maybe you can force them, but then I the future, if they see their parents who also go running and do sport and that, they also get used to seeing it in your own home, and it’s also good” (M3, FG5).
	Partnerships in activities	Family activities	“I have participated in mountain bike competitions and now I try to get my daughter to go out with me on the bike” (F8, FG6). “...Specifically, I do races, my girls come with me, the atmosphere of the day before, then the race, they get to know people, they get to know other children whose parents also race” (F6, FG6). “We do physical activity every day, we ride bicycles, we try... we also ride horses and we like to go for a walk, to run in the parks” (F7, FG4).
		Weekend activities	“And then maybe on a Saturday or Sunday you take them to the sports city to play football or basketball or something like that” (M1, FG5). “And on the weekends we go out with him on the bike and also on skates or go for a walk in the countryside, we like to go out” (M3, FG5).
		Fathers encourage physical activity and active play	“He does a lot of sports with his father, swimming, cycling, and I have noticed in him, in his conversations, that with his father he does activities, I mean with me he does activities where emotions are much more involved” (M4, FG2).
	Support from parents	Respect for the choice of children	“I don’t care, I like all of them, but I always look for something that motivates them, and that they really enjoy and have a good time with [...] so for the moment, they are happy with basketball” (F2, FG1). “...I like them to do some sport, but, as they said, something that they like, that they enjoy doing it so that they don’t get tired and that it is not something imposed by the father or the mother” (M1, FG1).
		Promote children’s autonomy	“...I leave him some time to decide what he wants to do, if he wants to play, if he wants to watch a bit of television, a bit of tablet and then early, at half past nine or so, he is in bed” (M4, FG2). “The mothers listen to them first, and depending on what the children say and we see more or less what they want, that’s how we do it” (M2, FG8).
	Value placed on physical activity by parents	Physical activity is important	“I also think it is important to practice sport, children are more active, with more energy” (M1, FG3).
		Physical health benefits	“I like my children to do sport because it is also good for them, it also takes away a bit of adrenaline, and I don’t know, it’s good for their health” (M3, FG5). “We are very aware of his weight, I always weigh him and tell him that he can’t get fat, that he has to move, that this is very good for him, for his health” (M4, FG4).
		Psychological benefits	“The stress of after school activities, school, the continuous school day. Well, I think that sport, just as it helps us to disconnect, it helps them too” (M4, FG6). “You see, I think that sport is good for them, and not only for children, in general it is good for the brain” (M2, FG5).
		Social benefits	“I also think that it is also very good for them, playing with other children, companionship, learning to share with other children, playing in new situations” (M3, FG5). “...Coordination and sports are more important, where they learn the values of companionship and so on. They are the ones they have to learn at that age” (F3, FG1).
		Parents limit screen viewing time	“...Even if they are cartoons, that are didactic, or activities that are healthy for them, but... then with... well, a bit of everything, a bit of physical activity, not just watching TV or that, of tablets and new technologies, which is fine, but in the right measure” (M3, FG7).
Positive influence of friends		Choice of after school activities their friends do	“...If someone told him that he is going to join the football school, he will also want to join because he is his friend” (M1, FG5).
		Preferences for going out to play with friends	“If he’s alone, he usually plays a board game, or a game of Play Station or something like that. But if he is with other people, he’s not attracted to those games, he likes to play tag, or play with the ball or things like that” (M4, FG3). “These days when the weather is nice, he is eager to see the door open and see the girl in front of him to run out on the street” (M3, FG2).
		Having siblings of similar ages stimulates play	“She is a very active girl and is always playing with her brother” (M1, FG3).
School factors promoting physical activity	Built environment of the school	Playground can encourage physical activity	“There is a large playground, there is a lot of... they should put, they could do more different activities at recess, invent new games for the children, I don’t know, things like that to motivate them not to sit down” (M2, FG3).
		Existence of adequate spaces and materials	“The playground, yes, and the courts that are looked after and in good condition, so that the children can play. They should be provided with balls and things, but of course, that’s where they have physical education classes” (M3, FG8).
	Teacher modelling	Involved and motivated teachers	“Recess, teachers who want to get involved, organize it a bit, so that they are not throwing stones at recess time, sitting on one side without knowing what to do, so they organize it a bit and they are delighted” (F2, FG6).
		Teachers who encourage movement in class	“He talks to them about the importance, my son tells me a lot about what V tells them, V talks to them about how important it is, he tells them ’he who moves his legs moves his heart!’ that’s why they are always moving their legs [laughs], it’s fundamental” (M4, FG2).
Household and neighborhood factors that encouraged physical activity		Large houses and single family dwellings	“If I have a big house, apart from the fact that I don’t put any limitations on them. If the little one wants to move the bike to the living room, I leave him with the bike in the living room. When they’re older, I’ll have it more organized. Then they play all over the house, they have a yard, they have a swimming pool and downstairs they also have a big place, so if they want to ride their bikes or whatever they want” (M1, GF3).
		Houses with terrace or garden	“I do have a big courtyard, where they can run, play and now in this weather they usually go outside. Yesterday they were outside all afternoon, but of course, they exercise” (M5, FG3).
		Neighborhoods with common areas	“We live in a housing estate and as soon as the weather is good, they go out with their racquets, they go out with their balls and they spend an hour playing by themselves” (F3, FG1). “...It is also easier when the environment makes it easier for you, for example, now that we live in a housing estate, it is very easy. So they can go outside and run around, play..." (M5, FG1).
		The street where they live allows them to go out to play	“I’m lucky to live on a street that is pedestrianized so you can go out and play there” (M4, FG3).

Abbreviations: M = Mother; F = Father; FG = Focus Group. Distinction of FG by rural or urban setting: Rural area: FG3, FG4, FG7, and FG8; Urban area: FG1, FG2, FG5, and FG6.

**Table 10:** Mesosystem facilitators for physical activity among schoolchildren.

Mesosystem			
Categories	Subcategories	Code	Parents’ Verbalizations
Non-school periods and free time		Children are more active on holidays	“...But maybe in the summer they do more physical activity. They go with their grandparents to the beach and there they spend the whole day playing football, in the swimming pool, in the... whatever. Basketball, tennis” (F2, FG1).
		Children are more active during weekends	“Then at weekends in the village he has the bike, and there too, but at his own free will” (M4, FG1). “I mean, at weekends, of course you don’t have to follow the routine, because of course, the children have their school and you have to respect that, you have to... so if you follow it, but at weekends you have more freedom, I mean, we are going to get up early and go to this place, we are going to do this, they don’t have school, and of course the parents don’t have to work, you have more free time, so you can do a lot more activities with them” (M3, FG2).
The school as a setting that transmits values		School is important for promoting physical activity	“It is very important, but everyone, even the members of the child’s family, it is very important that the child is taught sport” (M4, FG2). “I think it is very important, I mean, what we are, what we said before, children, the younger they are, they are sponges, in terms of, in terms of learning languages, everything. So, if you, if they have a good sporting education when they are young, I don’t know what they are taught, psychomotor skills, all these things, you can teach a young child to swim and they will learn better than if you try to learn when they are older, because you have already picked up habits and changing those habits, at least for me it is more difficult to change them than learning from scratch” (M1, FG5).

Abbreviations: M = Mother; F = Father; FG = Focus Group. Distinction of FG by rural or urban setting: Rural area: FG3, FG4, FG7, and FG8; Urban area: FG1, FG2, FG5, and FG6.

**Table 11:** Exosystem facilitators for physical activity among schoolchildren.

Exosystem			
Categories	Subcategories	Code	Parents’ Verbalizations
Community facilitators and the physical and built environment promoting physical activity		Good weather	“Every day of the month he swims in the summer, just when school finishes in June, and then he spends the whole summer, every day in the swimming pool, so summer is when he does sport seriously, every day, a lot” (M2, FG2). “In the summer, when it’s hot, they come home from school, they eat, they go out again, they come home, they cool down a bit, they eat again and go to the park” (M2, FG5).
	Factors in the public and community space that promote physical activity	Living in a rural area	“It’s different living in a city than living in a town, that’s for sure. Because in a city, well, first you go everywhere by car, with public transport, you don’t walk, unless you go... you do a separate activity. In the village you can walk anywhere, to school, to activities, to go shopping. So that’s something different from the city” (M4, FG7).
		Living in a small town	“...Well, in a city like Cuenca, which is small, where you can walk to many places, where you can see your schoolmates or friends because you meet them in the street, well, that also makes it much easier, to have that environment, what she said about living in Madrid, where they leave school, they have to take the metro, they have to take a bus, so the environment changes a lot, of course” (F1, FG1). “Then living in a, in a small town, where everything is close, I think that.... well, it’s positive in that, it’s not like in a city where you have to take the metro or take the train, bus and all that. I think that here you have everything next to home” (M1, FG5).
		Wide range of activities and infrastructures in urban areas	“Living in a small city, as he said, also the sports facilities that you can find. You can’t have sports facilities for everything, like in a big city. In a big city, you want to practice water polo and here you know for example that you have a swimming pool, but it’s for swimming” (F1, FG1).
		Existence of parks and gardens close to home	“So if you are lucky enough to live near a park or to be able to have one nearby, they go out on their own and run, of course” (M5, FG1).
Organization of after school activities related to sport and physical activity.		Involvement of the parents’ association in the organization of after school activities	“...They have n parents’ association that is very involved and all that, and here you can see that they are more relaxed” (M1, FG2). “Their role is to organize everything, both sports and after school activities, or... to organize everything, and they plan the whole year” (M4, FG5).

Abbreviations: M = Mother; F = Father; FG = Focus Group. Distinction of FG by rural or urban setting: Rural area: FG3, FG4, FG7, and FG8; Urban area: FG1, FG2, FG5, and FG6.

**Table 12:** Macrosystem facilitators for physical activity among schoolchildren.

Macrosystem			
Categories	Subcategories	Code	Parents’ verbalizations
Facilitators of the socio cultural and political context		Physical activity is in fashion	“I do think that fashion influences everything, just like fashion in clothes, fashion in sport, because before, nobody ran, and now everybody runs like fools, they run and run” (F1, FG7).
		Free or subsidized activities and venues	"Because the activities are free, for example. For example, the children here the activities are free, so they do the activities there, they come here to play whatever they do here” (M4, FG5).

Abbreviations: M = Mother; F = Father; FG = Focus Group. Distinction of FG by rural or urban setting: Rural area: FG3, FG4, FG7, and FG8; Urban area: FG1, FG2, FG5, and FG6.

## Data Availability

The study did not report any data.
